# On the development of new cosine-based probabilistic methods with applications to univariate and bivariate analyses of the wind speed energy

**DOI:** 10.1016/j.heliyon.2023.e21482

**Published:** 2023-10-26

**Authors:** Badr Alnssyan, Mohammed Ahmed Alomair

**Affiliations:** a Unit of Scientific Research, Applied College, Qassim University, Buraydah 51452, Saudi Arabia; b Department of Quantitative Methods, School of Business, King Faisal University, Al-Ahsa 31982, Saudi Arabia

**Keywords:** Weibull distribution, Cosine function, Univariate and bivariate distributions, Wind speed, Energy, Statistical modeling, Spain

## Abstract

So far in the literature, a number of probability distributions have been successfully implemented for analyzing the wind speed and energy data sets. However, there is no published work on modeling and analyzing the wind speed and energy data sets with probability distributions that are introduced using trigonometric functions. In the existing literature, there is also a lack of studies on implementing the bivariate trigonometric-based probability distributions for modeling the wind speed and energy data sets. In this paper, we take up a meaningful effort to cover these interesting research gaps. Thus, we first incorporate a cosine function and introduce a new univariate probability distributional method, namely, a univariate modified cosine-*G* (UMC-*G*) family. Using the UMC-*G* method, a new probability distribution called a univariate modified cosine-Weibull (UMC-Weibull) distribution is studied. We apply the UMC-Weibull distribution for analyzing the wind energy data set taken from the weather station at Sotavento Galicia, Spain. Furthermore, we also introduce a bivariate version of the UMC-*G* method using the Farlie–Gumble–Morgenstern copula approach. The proposed bivariate distributional method is called a bivariate modified cosine-*G* (BMC-*G*) family. A special member of the BMC-*G* distributions called a bivariate modified cosine-Weibull (BMC-Weibull) distribution is introduced. We apply the BMC-Weibull distribution for analyzing the bivariate data set representing the wind speed and energy taken from the weather station at Sotavento Galicia. Using different statistical tools, we observe that the UMC-Weibull and BMC-Weibull are the best-suited models for analyzing the wind speed and energy data sets.

## 1 Introduction

Among the possible sources of energy, wind is one of the most useful ways of generating energy, termed wind energy. Wind energy provides many environmental and economic advantages as compared to other sources such as fossil fuel-based energy, which badly pollutes the atmosphere. Since wind speed is the most important and significant parameter of generating wind energy. Therefore, an accurate determination and the best possible selection of probability distributions for modeling and estimating the wind speed energy are very important [1], [2], [3], [4].

Wind speed as an energy source is extremely valuable. Therefore, in recent years, there has been an increased and considerable interest in developing suitable probability distributions for predicting and modeling the energy output of wind energy conversion systems [5]. In this regard, several new probability distributions are developed and implemented successfully for modeling the wind speed energy data sets. For example, Kantar et al. [6] introduced a modified version of the Lindley distribution called the extended generalized Lindley (EGL) distribution. The cumulative distribution function (CDF) of the EGL distribution is given by

$$G\left(y\right)=1-\frac{\left(1+\lambda {\left(1+\eta y\right)}^{\theta }\right)}{\lambda +1}e^{\lambda -\lambda {\left(1+\eta y\right)}^{\theta }},\;\;\;\;\;\;y,\lambda ,\eta ,\theta \in R^{+}.$$

They implemented the EGL distribution for analyzing the wind speed data taken from different locations in Turkey.

Jia et al. [7] proposed another new probability distribution called the Topp-Leone Lindley (TLL) distribution with CDF

$$G\left(y\right)={\left(1-{\left(\frac{1+\lambda y+\lambda }{\lambda +1}e^{-\lambda y}\right)}^{2}\right)}^{\theta },\;\;\;\;\;\;y,\lambda ,\theta \in R^{+}.$$

They used the TLL distribution for analyzing the long-term measured wind speed data taken from different wind stations in China.

Ul-Haq et al. [8] introduced a new version of the power Lindley distribution using the alpha power transformation of Elbatal et al. [9]. They called the proposed model a new alpha power transformed power Lindley (NAPTPL) distribution. The CDF of the NAPTPL distribution is

$$G\left(y\right)=\frac{\left(1-\left(1+\frac{\lambda y^{\tau }}{\lambda +1}\right)e^{-\lambda y^{\tau }}\right)\alpha^{\left(1-\left(1+\frac{\lambda y^{\tau }}{\lambda +1}\right)e^{-\lambda y^{\tau }}\right)}}{\alpha },\;\;\;\;\;\;y,\lambda ,\tau ,\alpha \in R^{+},\alpha \neq 1.$$

The NAPTPL distribution was implemented for modeling the wind speed data taken from five different locations in Pakistan including Haripur, Gwadar, Quetta, Bahawalpur, and Peshawar. For more studies about the development of probability distributions for modeling the wind speed energy data sets, we refer to [10], [11], [12], [13], [14], [15], [16], [17].

As we briefly discussed above that a series of probability distributions have been introduced and applied for analyzing the wind speed energy data sets. However, according to our deep search of the literature, we did not find any published paper related to wind speed energy data modeling using trigonometric-based probability distributions. Secondly, there is also no published paper related to wind speed and energy data modeling using bivariate trigonometric-based probability distributions. In this paper, we attempt to produce useful efforts to cover these two amazing and interesting research gaps. In the first attempt, we develop a univariate trigonometric-based distributional method for modeling the wind energy data. In the second attempt, we develop a bivariate trigonometric-based distributional method for analyzing the wind speed and energy data.

The rest parts of this paper are carried out as follows: a univariate trigonometric-based distributional method is introduced in Section 2. Furthermore, a special member of the univariate distributional method is also discussed. In Section 3, the proposed bivariate distributional method is provided. Its special member is also discussed in detail. In Section 4, two practical applications representing wind speed and energy data sets are analyzed. Some concluding remarks are provided in Section 5.

## 2 A univariate modified cosine-*G* method and its special case

Recent development in distribution theory via incorporating trigonometric functions has received considerable attention [18], [19], [20]. In this section, we present our first proposal by introducing a new distributional method, namely, a univariate modified cosine-*G* (UMC-*G*) family of distributions. As its name suggests, the proposed method is obtained by incorporating a cosine function. Using the first proposal, we consider a special case to illustrate the applications of the UMC-*G* distributions.

### 2.1 A univariate modified cosine-*G* method

Assume *Y* is a UMC-*G* distributed random variable defined on R. The CDF of *Y*, say F(y), has the following form

(1) $$F\left(y\right)=\frac{\gamma \cos\left[\frac{\pi }{2}\overset{¯}{G}\left(y\right)\right]}{\gamma -1+\cos\left[\frac{\pi }{2}\overset{¯}{G}\left(y\right)\right]},\;\;\;\;\;\;y\in R,\gamma >1,$$

where $\overset{¯}{G}\left(y\right)=1-G\left(y\right)$ and *γ* is an additional parameter.

The probability distribution function (PDF) of the UMC-*G* models, say f(y), is defined as

$$f\left(y\right)=\frac{\pi \gamma \left(\gamma -1\right)g\left(y\right)\sin\left[\frac{\pi }{2}\overset{¯}{G}\left(y\right)\right]}{2{\left(\gamma -1+\cos\left[\frac{\pi }{2}\overset{¯}{G}\left(y\right)\right]\right)}^{2}},\;\;\;\;\;\;y\in R,\gamma >1,$$

where $\frac{d}{dy}G\left(y\right)=g\left(y\right)$.

The survival function (SF) of *Y*, say S(y), is given by

$$S\left(y\right)=\frac{\left(\gamma -1\right)\left(1-\cos\left[\frac{\pi }{2}\overset{¯}{G}\left(y\right)\right]\right)}{\gamma -1+\cos\left[\frac{\pi }{2}\overset{¯}{G}\left(y\right)\right]},\;\;\;\;\;\;y\in R,\gamma >1.$$

Whereas, the hazard function (HF) h(y) and cumulative HF (CHF) H(y) of the MS-*G* models are given, respectively, by

$$h\left(y\right)=\frac{\pi \gamma g\left(y\right)\sin\left[\frac{\pi }{2}\overset{¯}{G}\left(y\right)\right]}{2\left(1-\cos\left[\frac{\pi }{2}\overset{¯}{G}\left(y\right)\right]\right)\left(\gamma -1+\cos\left[\frac{\pi }{2}\overset{¯}{G}\left(y\right)\right]\right)},\;\;\;\;\;\;y\in R,\gamma >1,$$

and

$$H\left(y\right)=-\log\left(\frac{\left(\gamma -1\right)\left(1-\cos\left[\frac{\pi }{2}\overset{¯}{G}\left(y\right)\right]\right)}{\gamma -1+\cos\left[\frac{\pi }{2}\overset{¯}{G}\left(y\right)\right]}\right),\;\;\;\;\;\;y\in R,\gamma >1.$$

In the next subsection, we discuss a special case of the UMC-*G* distributional method, namely, a univariate modified cosine-Weibull (UMC-Weibull) distribution.

### 2.2 A special case of the UMC-*G* method

In this section, we define the basic functions of the special member of the UMC-*G* method. For this purpose, we consider the Weibull distribution with shape *τ* and scale *β* parameters taken as a baseline model.

Assume *Y* is a Weibull distributed random variable defined on $R^{+}$. The CDF G(y) and PDF g(y) of *Y* are given, respectively, by

(2) $$G\left(y\right)=1-e^{-\beta y^{\tau }},\;\;\;\;\;\;\;\;\;\;y\geq 0,$$

and

$$g\left(y\right)=\tau \beta y^{\tau -1}e^{-\beta y^{\tau }},\;\;\;\;\;\;\;\;\;\;y>0.$$

Using Eq. (2) in Eq. (1), we obtain the CDF and SF of the UMC-Weibull distribution given by

$$F\left(y\right)=\frac{\gamma \cos\left[\frac{\pi }{2}e^{-\beta y^{\tau }}\right]}{\gamma -1+\cos\left[\frac{\pi }{2}e^{-\beta y^{\tau }}\right]},\;\;\;\;\;\;y\in R^{+},\gamma >1,$$

and

(3) $$S\left(y\right)=\frac{\left(\gamma -1\right)\left(1-\cos\left[\frac{\pi }{2}e^{-\beta y^{\tau }}\right]\right)}{\gamma -1+\cos\left[\frac{\pi }{2}e^{-\beta y^{\tau }}\right]},\;\;\;\;\;\;y\in R^{+},\gamma >1,$$

respectively.

**Figure 1:**
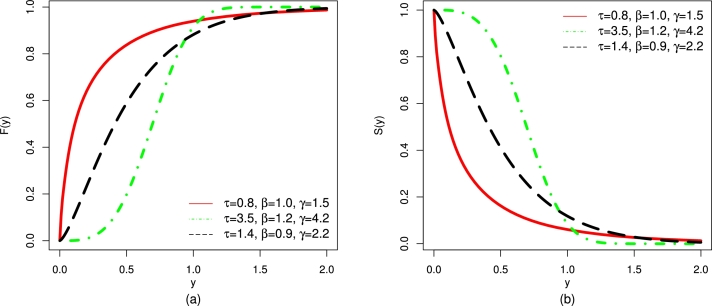
Graphical illustrations of the (a) CDF and (b) SF of the UMC-Weibull distribution.

For different values of τ,β, and *γ*, the CDF and SF plots of the UMC-Weibull distribution are provided in Fig. 1.

The PDF of the UMC-Weibull distribution is

(4) $$f\left(y\right)=\frac{\pi \gamma \left(\gamma -1\right)\tau \beta y^{\tau -1}e^{-\beta y^{\tau }}\sin\left[\frac{\pi }{2}e^{-\beta y^{\tau }}\right]}{2{\left(\gamma -1+\cos\left[\frac{\pi }{2}e^{-\beta y^{\tau }}\right]\right)}^{2}},\;\;\;\;\;\;y\in R,\gamma >1.$$

**Figure 2:**
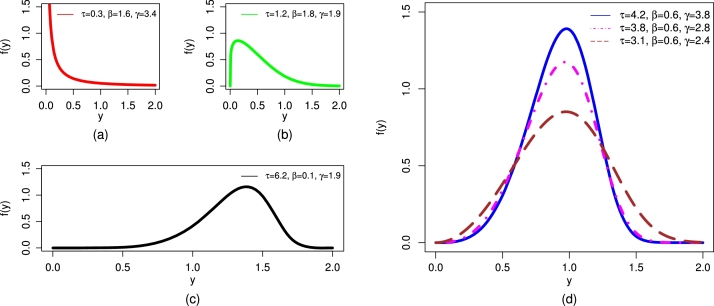
Different illustrations of the PDF of the UMC-Weibull distribution, including (a) decreasing, (b) righted-skewed, (c) left-skewed, and (d) symmetrical.

For various selected values of τ,β, and *γ*, different PDF plots of the UMC-Weibull distribution are illustrated in Fig. 2.

By taking the ratio of Eq. (3) and Eq. (4), we get the HF of the UMC-Weibull distribution given by

$$h\left(y\right)=\frac{\pi \gamma \tau \beta y^{\tau -1}e^{-\beta y^{\tau }}\sin\left[\frac{\pi }{2}e^{-\beta y^{\tau }}\right]}{2\left(1-\cos\left[\frac{\pi }{2}e^{-\beta y^{\tau }}\right]\right)\left(\gamma -1+\cos\left[\frac{\pi }{2}e^{-\beta y^{\tau }}\right]\right)},\;\;\;\;\;\;y\in R,\gamma >1.$$

**Figure 3:**
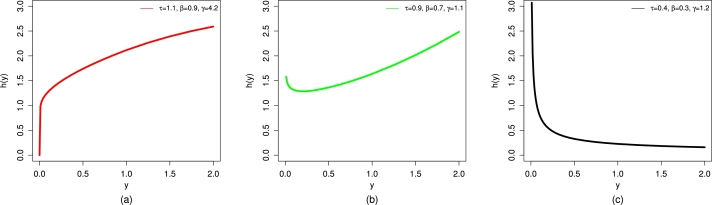
Different illustrations of the HF of the UMC-Weibull distribution, including (a) increasing, (b) bathtub, and (c) decreasing.

For different chosen values of τ,β, and *γ*, the HF plots of the UMC-Weibull distribution are visualized in Fig. 3.

Furthermore, the cumulative hazard function (CHF) and reverse hazard function (RHF) of the UMC-Weibull distribution are given by

$$H\left(y\right)=-\log\left(\frac{\left(\gamma -1\right)\left(1-\cos\left[\frac{\pi }{2}e^{-\beta y^{\tau }}\right]\right)}{\gamma -1+\cos\left[\frac{\pi }{2}e^{-\beta y^{\tau }}\right]}\right),\;\;\;\;\;\;y\in R^{+},\gamma >1,$$

and

$$R\left(y\right)=\frac{\pi \left(\gamma -1\right)\tau \beta y^{\tau -1}e^{-\beta y^{\tau }}\sin\left[\frac{\pi }{2}e^{-\beta y^{\tau }}\right]}{2\cos\left[\frac{\pi }{2}e^{-\beta y^{\tau }}\right]\left(\gamma -1+\cos\left[\frac{\pi }{2}e^{-\beta y^{\tau }}\right]\right)},\;\;\;\;\;\;y\in R,\gamma >1,$$

respectively.

**Figure 4:**
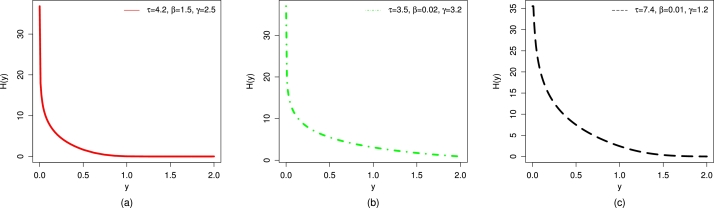
Graphical illustrations of the (a) CHF with red color, (b) CHF with green color, and (c) CHF with black color of the UMC-Weibull distribution.

For different selected values of τ,β, and *γ*, the plots of the CHF of the UMC-Weibull distribution are presented in Fig. 4.

By incorporating the UMC-*G* distributional method, a series of new modified distributions can be obtained. In this regard, some special members of the UMC-*G* distributional method are presented in Table 1.

**Table 1:** Some special members of the UMC-*G* distributional method.

S. No.	Baseline Model	Baseline SF $\overset{¯}{G}\left(y\right)$	Proposed CDF F(y)
1	Rayleigh	$e^{-\beta y^{2}}$	$\frac{\gamma \cos\left[\frac{\pi }{2}e^{-\beta y^{2}}\right]}{\gamma -1+\cos\left[\frac{\pi }{2}e^{-\beta y^{2}}\right]}$
2	Exponential	*e*^−*βy*^	$\frac{\gamma \cos\left[\frac{\pi }{2}e^{-\beta y}\right]}{\gamma -1+\cos\left[\frac{\pi }{2}e^{-\beta y}\right]}$
3	Lomax	${\left(1+\theta y\right)}^{-\alpha }$	$\frac{\gamma \cos\left[\frac{\pi }{2}{\left(1+\theta y\right)}^{-\alpha }\right]}{\gamma -1+\cos\left[\frac{\pi }{2}{\left(1+\theta y\right)}^{-\alpha }\right]}$
4	Linear failure rate	$e^{-\left(\beta y^{2}+\eta y\right)}$	$\frac{\gamma \cos\left[\frac{\pi }{2}e^{-\left(\beta y^{2}+\eta y\right)}\right]}{\gamma -1+\cos\left[\frac{\pi }{2}e^{-\left(\beta y^{2}+\eta y\right)}\right]}$
5	Modified Weibull	$e^{-\left(\beta y^{\tau }+\eta y\right)}$	$\frac{\gamma \cos\left[\frac{\pi }{2}e^{-\left(\beta y^{\tau }+\eta y\right)}\right]}{\gamma -1+\cos\left[\frac{\pi }{2}e^{-\left(\beta y^{\tau }+\eta y\right)}\right]}$
6	Uniform	(λ−y)/λ	$\frac{\gamma \cos\left[\frac{\pi }{2}\left(\lambda -y\right)/\lambda \right]}{\gamma -1+\cos\left[\frac{\pi }{2}\left(\lambda -y\right)/\lambda \right]}$
7	Burr	${\left(1+y^{\sigma }\right)}^{-\phi }$	$\frac{\gamma \cos\left[\frac{\pi }{2}{\left(1+y^{\sigma }\right)}^{-\phi }\right]}{\gamma -1+\cos\left[\frac{\pi }{2}{\left(1+y^{\sigma }\right)}^{-\phi }\right]}$
8	Kumaraswamy	${\left(1-y^{a}\right)}^{b}$	$\frac{\gamma \cos\left[\frac{\pi }{2}{\left(1-y^{a}\right)}^{b}\right]}{\gamma -1+\cos\left[\frac{\pi }{2}{\left(1-y^{a}\right)}^{b}\right]}$

## 3 A bivariate modified cosine-*G* method and its special case

When there is a dependency between two variables, in such cases the bivariate distributions provide satisfactory results. The bivariate distributions have been successfully implemented for modeling real-life phenomena in reliability, hydrological, drought, sport, and many others. Due to satisfactory results of the bivariate distributions in these sectors, researchers have shown an increased interest in developing new bivariate distributions, see for example [21], [22], [23], [24], [25].

In the statistical literature, the Farlie-Gumbel-Morgenstern (FGM) has proven to be the most prominent approach for generating bivariate distributions. The introduction of the FGM distributions years back to mid 20th century due to Morgenstern [26]. Later, a multivariate version of the FGM method was studied by Farlie [27].

Assume $Y_{1}$ has a CDF $F\left(y_{1}\right)$ and $Y_{2}$ has a CDF $F\left(y_{2}\right)$, then, the joint CDF (i.e., bivariate CDF) of the FGM distributions, say $F\left(y_{1},y_{2}\right)$, is given by

(5) $$F\left(y_{1},y_{2}\right)=F\left(y_{1}\right)F\left(y_{2}\right)\left[1+\delta \overset{¯}{F}\left(y_{1}\right)\overset{¯}{F}\left(y_{2}\right)\right],$$

where $-1\leq \delta \leq 1,\overset{¯}{F}\left(y_{1}\right)=1-F\left(y_{1}\right)$, and $\overset{¯}{F}\left(y_{2}\right)=1-F\left(y_{2}\right)$. When, δ=0, Eq. (5) reduces to

$$F\left(y_{1},y_{2}\right)=F\left(y_{1}\right)F\left(y_{2}\right).$$

Corresponding to Eq. (5), the bivariate PDF $f\left(y_{1},y_{2}\right)$ of the FGM of distributions is given by

$$f\left(y_{1},y_{2}\right)=f\left(y_{1}\right)f\left(y_{2}\right)\left(1+\delta \left[1-2F\left(y_{1}\right)\right]\left[1-2F\left(y_{2}\right)\right]\right).$$

In the next section, we use the FGM distributions approach to introduce the bivariate version of the UMC-*G* distributions, namely, a bivariate modified cosine-*G* (BMC-*G*) distributions.

### 3.1 A bivariate modified cosine-*G* method

Assume $Y_{1}$ follows the UMC-*G* models with CDF, say $F\left(y_{1}\right)$, is given by

(6) $$F\left(y_{1}\right)=\frac{\gamma_{1}\cos\left[\frac{\pi }{2}\overset{¯}{G}\left(y_{1}\right)\right]}{\gamma_{1}-1+\cos\left[\frac{\pi }{2}\overset{¯}{G}\left(y_{1}\right)\right]},\;\;\;\;\;\;y_{1}\in R,\gamma_{1}>1,$$

and PDF $f\left(y_{1}\right)$ given by

$$f\left(y_{1}\right)=\frac{\pi \gamma_{1}\left(\gamma_{1}-1\right)g\left(y_{1}\right)\sin\left[\frac{\pi }{2}\overset{¯}{G}\left(y_{1}\right)\right]}{2{\left(\gamma_{1}-1+\cos\left[\frac{\pi }{2}\overset{¯}{G}\left(y_{1}\right)\right]\right)}^{2}},\;\;\;\;\;\;y_{1}\in R,\gamma_{1}>1,$$

Now, assume $Y_{2}$ also follows the UMC-*G* models with CDF, say $F\left(y_{2}\right)$, is given by

(7) $$F\left(y_{2}\right)=\frac{\gamma_{2}\cos\left[\frac{\pi }{2}\overset{¯}{G}\left(y_{2}\right)\right]}{\gamma_{2}-1+\cos\left[\frac{\pi }{2}\overset{¯}{G}\left(y_{2}\right)\right]},\;\;\;\;\;\;y_{2}\in R,\gamma_{2}>1,$$

and PDF $f\left(y_{2}\right)$ given by

$$f\left(y_{2}\right)=\frac{\pi \gamma_{2}\left(\gamma_{2}-1\right)g\left(y_{2}\right)\sin\left[\frac{\pi }{2}\overset{¯}{G}\left(y_{2}\right)\right]}{2{\left(\gamma_{2}-1+\cos\left[\frac{\pi }{2}\overset{¯}{G}\left(y_{2}\right)\right]\right)}^{2}},\;\;\;\;\;\;y_{2}\in R,\gamma_{2}>1.$$

Using Eq. (6) and Eq. (7) in Eq. (5), we get the CDF of the BMC-*G* distributions given by

(8) $$F\left(y_{1},y_{2}\right)=\frac{\gamma_{1}\cos\left[\frac{\pi }{2}\overset{¯}{G}\left(y_{1}\right)\right]}{\gamma_{1}-1+\cos\left[\frac{\pi }{2}\overset{¯}{G}\left(y_{1}\right)\right]}\frac{\gamma_{2}\cos\left[\frac{\pi }{2}\overset{¯}{G}\left(y_{2}\right)\right]}{\gamma_{2}-1+\cos\left[\frac{\pi }{2}\overset{¯}{G}\left(y_{2}\right)\right]}\left[1+\delta \left(\frac{\left(\gamma_{1}-1\right)\left(1-\cos\left[\frac{\pi }{2}\overset{¯}{G}\left(y_{1}\right)\right]\right)}{\gamma_{1}-1+\cos\left[\frac{\pi }{2}\overset{¯}{G}\left(y_{1}\right)\right]}\right)\left(\frac{\left(\gamma_{2}-1\right)\left(1-\cos\left[\frac{\pi }{2}\overset{¯}{G}\left(y_{2}\right)\right]\right)}{\gamma_{2}-1+\cos\left[\frac{\pi }{2}\overset{¯}{G}\left(y_{2}\right)\right]}\right)\right].$$

The PDF of the BMC-*G* distributions is given by

$$f\left(y_{1},y_{2}\right)=\frac{\pi \gamma_{1}\left(\gamma_{1}-1\right)g\left(y_{1}\right)\sin\left[\frac{\pi }{2}\overset{¯}{G}\left(y_{1}\right)\right]}{2{\left(\gamma_{1}-1+\cos\left[\frac{\pi }{2}\overset{¯}{G}\left(y_{1}\right)\right]\right)}^{2}}\frac{\pi \gamma_{2}\left(\gamma_{2}-1\right)g\left(y_{2}\right)\sin\left[\frac{\pi }{2}\overset{¯}{G}\left(y_{2}\right)\right]}{2{\left(\gamma_{2}-1+\cos\left[\frac{\pi }{2}\overset{¯}{G}\left(y_{2}\right)\right]\right)}^{2}}\left(1+\delta \left[1-2\frac{\gamma_{1}\cos\left[\frac{\pi }{2}\overset{¯}{G}\left(y_{1}\right)\right]}{\gamma_{1}-1+\cos\left[\frac{\pi }{2}\overset{¯}{G}\left(y_{1}\right)\right]}\right]\left[1-2\frac{\gamma_{2}\cos\left[\frac{\pi }{2}\overset{¯}{G}\left(y_{2}\right)\right]}{\gamma_{2}-1+\cos\left[\frac{\pi }{2}\overset{¯}{G}\left(y_{2}\right)\right]}\right]\right).$$

In the next subsection, we provide a special member of the BMC-*G* distributions, namely, a bivariate modified cosine-Weibull (BMC-Weibull) distribution.

### 3.2 A special case of the BMC-*G* method

In this section, we define a special member of the BMC-*G* method. For this purpose, we again consider the Weibull model as a baseline distribution.

Assume $Y_{1}$ follows the Weibull distribution having CDF $G\left(y_{1}\right)$ with shape parameter $\tau_{1}$ and scale parameter $\beta_{1}$. The CDF of $Y_{1}$ is given by

(9) $$G\left(y_{1}\right)=1-e^{-\beta_{1}y_{1}^{\tau_{1}}},$$

and PDF

$$g\left(y_{1}\right)=\tau_{1}\beta_{1}y_{1}^{\tau_{1}-1}e^{-\beta_{1}y_{1}^{\tau_{1}}}.$$

Now, assume $Y_{2}$ follows the Weibull distribution having CDF $G\left(y_{2}\right)$ with shape parameter $\tau_{2}$ and scale parameter $\beta_{2}$. The CDF of $Y_{2}$ is given by

(10) $$G\left(y_{2}\right)=1-e^{-\beta_{2}y_{2}^{\tau_{2}}},$$

and PDF

$$g\left(y_{2}\right)=\tau_{2}\beta_{2}y_{2}^{\tau_{2}-1}e^{-\beta_{2}y_{2}^{\tau_{2}}}.$$

Using Eq. (9) and Eq. (10) in Eq. (8), we get the CDF of the BMC-Weibull distribution given by

(11) $$F\left(y_{1},y_{2}\right)=\frac{\gamma_{1}\cos\left[\frac{\pi }{2}e^{-\beta_{1}y_{1}^{\tau_{1}}}\right]}{\gamma_{1}-1+\cos\left[\frac{\pi }{2}e^{-\beta_{1}y_{1}^{\tau_{1}}}\right]}\frac{\gamma_{2}\cos\left[\frac{\pi }{2}e^{-\beta_{2}y_{2}^{\tau_{2}}}\right]}{\gamma_{2}-1+\cos\left[\frac{\pi }{2}e^{-\beta_{2}y_{2}^{\tau_{2}}}\right]}\left[1+\delta \left(\frac{\left(\gamma_{1}-1\right)\left(1-\cos\left[\frac{\pi }{2}e^{-\beta_{1}y_{1}^{\tau_{1}}}\right]\right)}{\gamma_{1}-1+\cos\left[\frac{\pi }{2}e^{-\beta_{1}y_{1}^{\tau_{1}}}\right]}\right)\left(\frac{\left(\gamma_{2}-1\right)\left(1-\cos\left[\frac{\pi }{2}e^{-\beta_{2}y_{2}^{\tau_{2}}}\right]\right)}{\gamma_{2}-1+\cos\left[\frac{\pi }{2}e^{-\beta_{2}y_{2}^{\tau_{2}}}\right]}\right)\right],$$

and SF given by

$$S\left(y_{1},y_{2}\right)=1-\frac{\gamma_{1}\cos\left[\frac{\pi }{2}e^{-\beta_{1}y_{1}^{\tau_{1}}}\right]}{\gamma_{1}-1+\cos\left[\frac{\pi }{2}e^{-\beta_{1}y_{1}^{\tau_{1}}}\right]}\frac{\gamma_{2}\cos\left[\frac{\pi }{2}e^{-\beta_{2}y_{2}^{\tau_{2}}}\right]}{\gamma_{2}-1+\cos\left[\frac{\pi }{2}e^{-\beta_{2}y_{2}^{\tau_{2}}}\right]}\left[1+\delta \left(\frac{\left(\gamma_{1}-1\right)\left(1-\cos\left[\frac{\pi }{2}e^{-\beta_{1}y_{1}^{\tau_{1}}}\right]\right)}{\gamma_{1}-1+\cos\left[\frac{\pi }{2}e^{-\beta_{1}y_{1}^{\tau_{1}}}\right]}\right)\left(\frac{\left(\gamma_{2}-1\right)\left(1-\cos\left[\frac{\pi }{2}e^{-\beta_{2}y_{2}^{\tau_{2}}}\right]\right)}{\gamma_{2}-1+\cos\left[\frac{\pi }{2}e^{-\beta_{2}y_{2}^{\tau_{2}}}\right]}\right)\right].$$

**Figure 5:**
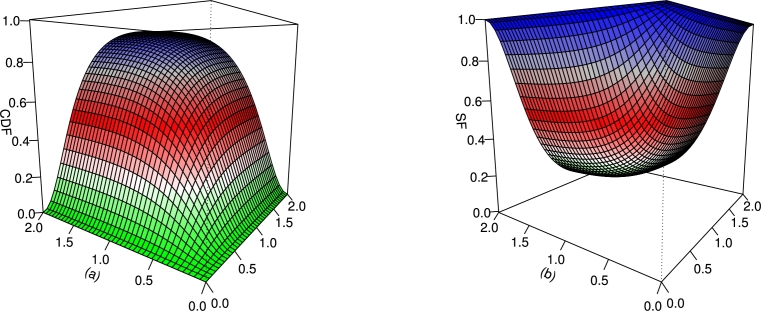
Graphical illustrations of the (a) CDF and (b) SF of the BMC-Weibull distribution for *τ*_1_ = *τ*_2_ = 2,*β*_1_ = *β*_2_ = 0.5,*γ*_1_ = *γ*_2_ = 1.2, and *δ* = 0.5.

**Figure 6:**
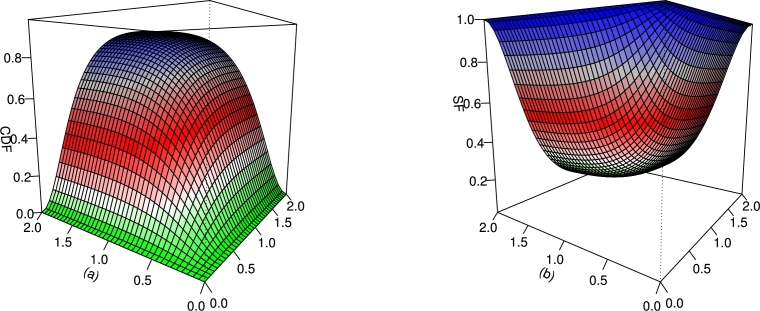
Graphical illustrations of the (a) CDF and (b) SF of the BMC-Weibull distribution for *τ*_1_ = *τ*_2_ = 2,*β*_1_ = *β*_2_ = 0.5,*γ*_1_ = *γ*_2_ = 1.2, and *δ* = −0.5.

For different values of $\tau_{1},\tau_{2},\beta_{1},\beta_{2},\gamma_{1},\gamma_{2}$ and *δ*, the plots for the CDF and SF of the BMC-Weibull distribution are presented in Figure 5, Figure 6. The plots in Figure 5, Figure 6 show that the BMC-Weibull distribution has a valid CDF, as the curves of these plots lie between zero and one.

Corresponding to Eq. (11), the PDF of the BMC-Weibull distribution is given by

$$f\left(y_{1},y_{2}\right)=\frac{\pi \gamma_{1}\left(\gamma_{1}-1\right)\tau_{1}\beta_{1}y_{1}^{\tau_{1}-1}e^{-\beta_{1}y_{1}^{\tau_{1}}}\sin\left[\frac{\pi }{2}e^{-\beta_{1}y_{1}^{\tau_{1}}}\right]}{2{\left(\gamma_{1}-1+\cos\left[\frac{\pi }{2}e^{-\beta_{1}y_{1}^{\tau_{1}}}\right]\right)}^{2}}\;\frac{\pi \gamma_{2}\left(\gamma_{2}-1\right)\tau_{2}\beta_{2}y_{2}^{\tau_{2}-1}e^{-\beta_{2}y_{2}^{\tau_{2}}}\sin\left[\frac{\pi }{2}e^{-\beta_{2}y_{2}^{\tau_{2}}}\right]}{2{\left(\gamma_{2}-1+\cos\left[\frac{\pi }{2}e^{-\beta_{2}y_{2}^{\tau_{2}}}\right]\right)}^{2}}\left(1+\delta \left[1-\frac{2\gamma_{1}\cos\left[\frac{\pi }{2}e^{-\beta_{2}y_{1}^{\tau_{2}}}\right]}{\gamma_{1}-1+\cos\left[\frac{\pi }{2}e^{-\beta_{2}y_{1}^{\tau_{2}}}\right]}\right]\left[1-\frac{2\gamma_{2}\cos\left[\frac{\pi }{2}e^{-\beta_{2}y_{2}^{\tau_{2}}}\right]}{\gamma_{2}-1+\cos\left[\frac{\pi }{2}e^{-\beta_{2}y_{2}^{\tau_{2}}}\right]}\right]\right).$$

**Figure 7:**
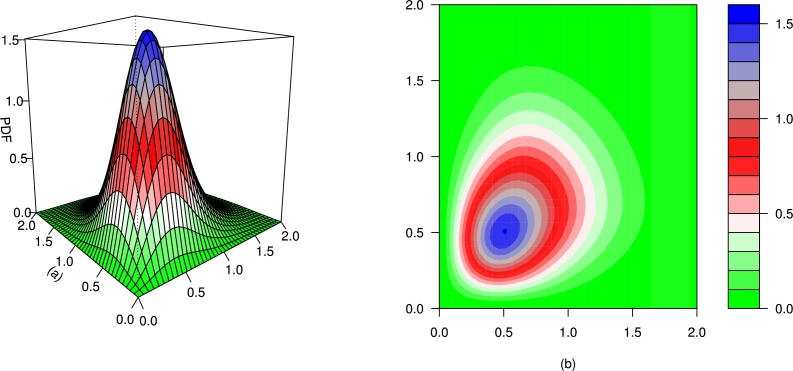
Graphical illustrations of the (a) PDF and (b) contour plot of the BMC-Weibull distribution for *τ*_1_ = *τ*_2_ = 2.5,*β*_1_ = *β*_2_ = 0.5,*γ*_1_ = *γ*_2_ = 1.5, and *δ* = 0.5.

**Figure 8:**
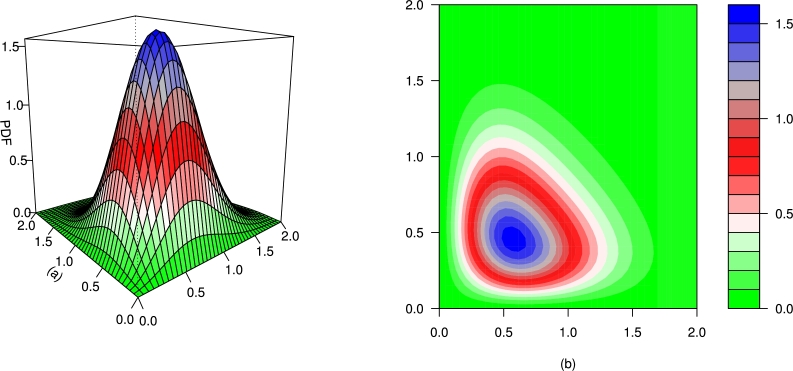
Graphical illustrations of the (a) PDF and (b) contour plot of the BMC-Weibull distribution for *τ*_1_ = *τ*_2_ = 2.5,*β*_1_ = *β*_2_ = 0.5,*γ*_1_ = *γ*_2_ = 1.5, and *δ* = −0.5.

**Figure 9:**
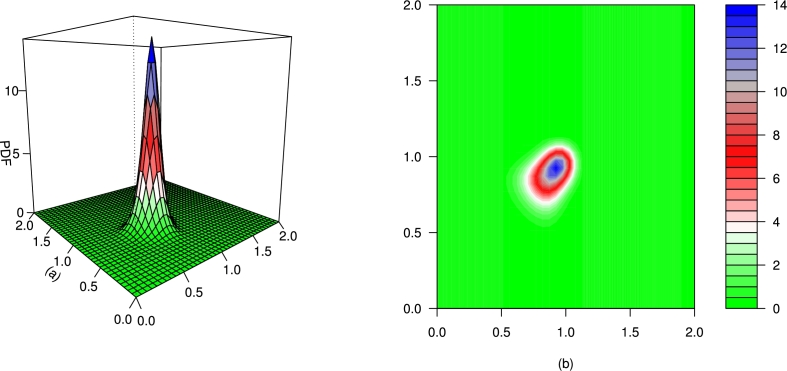
Graphical illustrations of the (a) PDF and (b) contour plot of the BMC-Weibull distribution for *τ*_1_ = *τ*_2_ = 9.2,*β*_1_ = *β*_2_ = 1,*γ*_1_ = *γ*_2_ = 3.5, and *δ* = 1.

**Figure 10:**
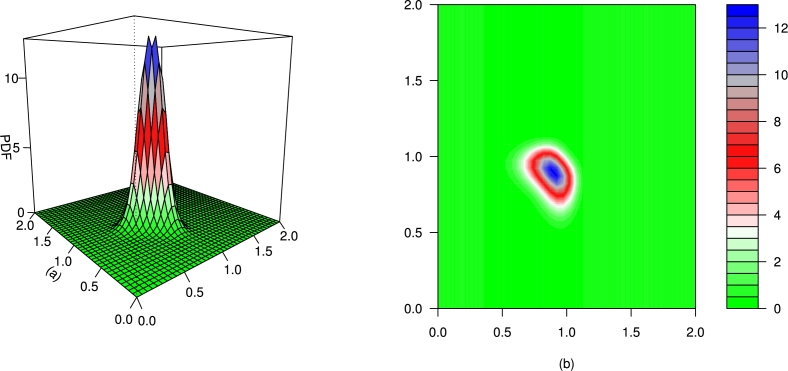
Graphical illustrations of the (a) PDF and (b) contour plot of the BMC-Weibull distribution for *τ*_1_ = *τ*_2_ = 9.2,*β*_1_ = *β*_2_ = 1,*γ*_1_ = *γ*_2_ = 3.5, and *δ* = −1.

**Figure 11:**
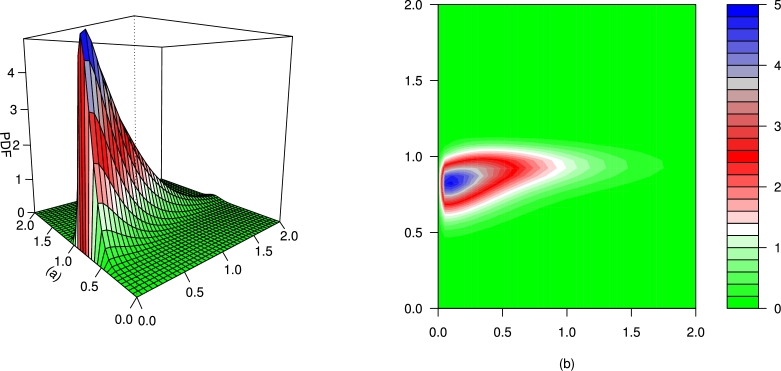
Graphical illustrations of the (a) PDF and (b) contour plot of the BMC-Weibull distribution for *τ*_1_ = 8.2,*τ*_2_ = 1.2,*β*_1_ = *β*_2_ = 1,*γ*_1_ = *γ*_2_ = 3.5, and *δ* = 1.

**Figure 12:**
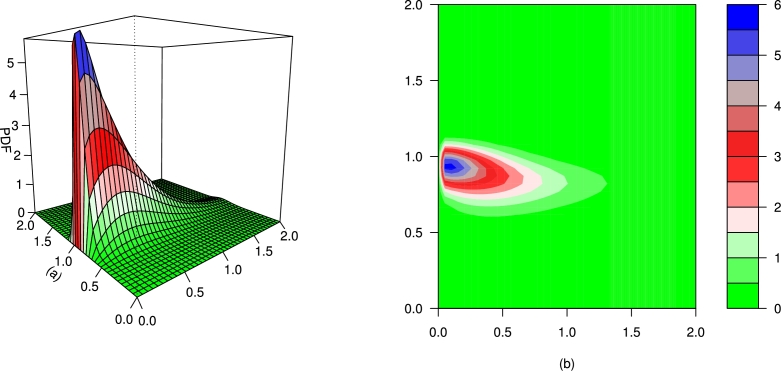
Graphical illustrations of the (a) PDF and (b) contour plot of the BMC-Weibulldistribution for *τ*_1_ = 1.2,*τ*_2_ = 8.2,*β*_1_ = *β*_2_ = 1,*γ*_1_ = *γ*_2_ = 3.5, and *δ* = 1.

For different values of $\tau_{1},\tau_{2},\beta_{1},\beta_{2},\gamma_{1},\gamma_{2}$ and *δ*, the plots for the PDF of the BMC-Weibull distribution are presented in Figure 7, Figure 8, Figure 9, Figure 10, Figure 11, Figure 12. The plots in Figure 7, Figure 8, Figure 9, Figure 10, Figure 11, Figure 12 show that the PDF of the BMC-Weibull distribution is unimodal and skewed to the right.

## 4 Univariate and bivariate analyses of the wind speed energy data sets

Basically, this section carries two aims and is divided into two subsections. In the first subsection, we apply the UMC-Weibull distribution for analyzing the wind energy data set. In the second subsection, we apply the BMC-Weibull distribution for analyzing the wind speed and energy data sets.

### 4.1 Univariate analysis of the wind energy data set

**Figure 13:**
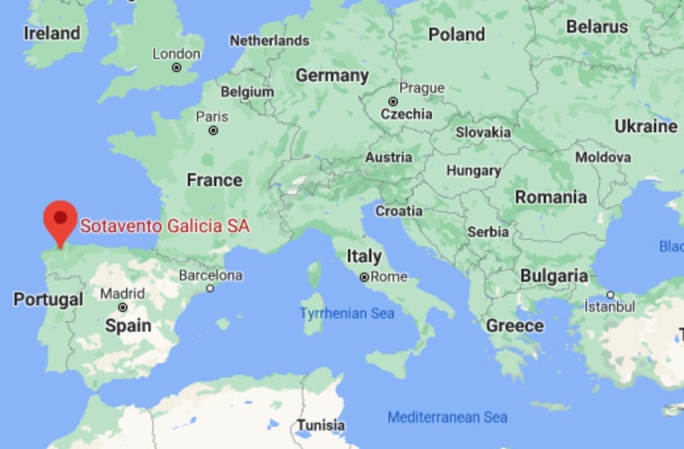
The location of the wind energy station at Sotavento Galicia, Spain.

As we discussed above that this subsection offers the illustration of the UMC-Weibull distribution using the wind energy data set. For this purpose, we consider a practical application representing the wind energy recorded hourly at the weather station of Sotavento Galicia, Spain. The wind energy data set was recorded on July 19, 2023. This data set is also available at: http://www.sotaventogalicia.com/en/technical-area/real-time-data/historical/. The Sotavento Galicia weather station is located at 43.3544° N and 7.8812° W; see Fig. 13.

**Figure 14:**
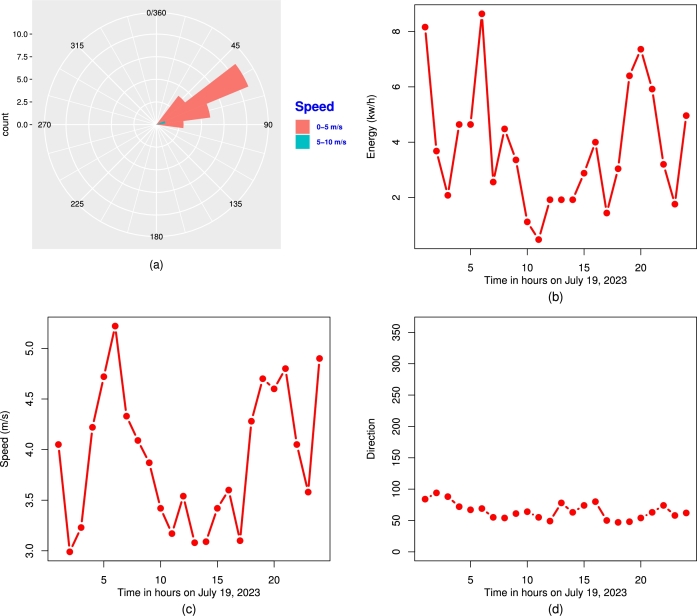
The visual illustration of the (a) wind rose, (b) energy, (c) wind speed, and (d) degrees of the wind.

**Figure 15:**
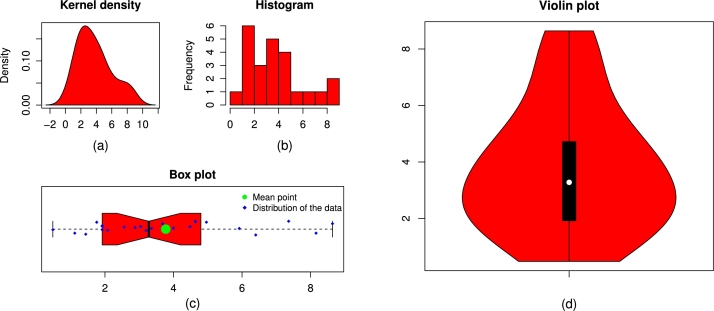
The plots of the (a) kernel density, (b) histogram, (c) box plot, and (d) violin plot of the wind energy data.

Some descriptive plots of the data set are presented in Fig. 14. These plots include the hourly (i) wind rose (top-left plot), (ii) produced energy (top-right plot), (iii) wind speed (m/s) (bottom-left plot), and (iv) degrees of the wind (bottom-right plot). Furthermore, the kernel density, histogram, box plot, and violin plots of the wing energy data are provided in Fig. 15.

Using the wind energy data set, we prove the applicability of the UMC-Weibull distribution by comparing its fitting power with some prominent probability distributions. The CDFs of these probability distributions are given by

• Flexible Weibull (F-Weibull) distribution of Bebbington et al. [28]
  $$G\left(y\right)=1-e^{-e^{\left(\beta y-\frac{\eta }{y}\right)}},\;\;\;\;\;\;\;\;\;\;y,\beta ,\eta \in R^{+},$$
• New modified flexible Weibull (NMF-Weibull) distribution of Ahmad et al. [29]
  $$G\left(y\right)=\frac{\lambda -\lambda e^{-e^{\left(\beta y-\frac{\eta }{y}\right)}}}{\lambda -e^{-e^{\left(\beta y-\frac{\eta }{y}\right)}}},\;\;\;\;\;\;\;\;\;\;y,\beta ,\eta \in R^{+},\lambda >1.$$
• New cotangent-Weibull (NCT-Weibull) distribution of Odhah et al. [30]
  $$G\left(y\right)=1-\frac{e^{-\beta y^{\tau }}}{e^{\cot\left(\frac{\pi }{2}e^{-\beta y^{\tau }}\right)}},\;\;\;\;\;\;\;\;\;\;y,\tau ,\beta \in R^{+}.$$
• Modified Weibull (M-Weibull) distribution of Sarhan and Zaindin [31]
  $$G\left(y\right)=1-e^{-\left(\beta y^{\tau }+\sigma y\right)},\;\;\;\;\;\;\;\;\;\;y,\tau ,\beta ,\sigma \in R^{+}.$$

Three statistical tests and the p-value are considered to compare the fitting results of the UMC-Weibull distribution and the above competing distributions. The statistical tests include

• The Cramér–von Mises test denoted by $W^{⁎}$ and is given by
  $$W^{⁎}=\sum_{i=1}^{n}{\left[\frac{2i-1}{2n}-G\left(y_{i}\right)\right]}^{2}+\frac{1}{12n}.$$
• The Anderson–Darling test expressed by $A^{⁎}$ and is calculated as
  $$A^{⁎}=-\frac{1}{n}\sum_{i=1}^{n}\left[\log\left\{1-G\left(y_{n-i+1}\right)\right\}+\log G\left(y_{i}\right)\right]\left(2i-1\right)-n.$$
• The Kolmogorov–Smirnov test represented by $KS^{⁎}$ and is obtained as
  $$KS^{⁎}=sup_{y}\left[G_{n}\left(y\right)-G\left(y\right)\right].$$

In the above formulas of the statistical tests, the terms $y_{i}$ and *n* represent, respectively, the $i^{th}$ observation and size of the data. The terms $G\left(y_{i}\right)$ and $G\left(y_{n-i+1}\right)$ represent the CDFs of the competing distribution corresponding to the $i^{th}$ and ${\left(n-i+1\right)}^{th}$ observations of the data, respectively. The $G_{n}\left(y\right)$ and G(y) represent the empirical CDF and CDF of the competing distribution, respectively. Whereas, $sup_{y}$ represents the set of differences between $G_{n}\left(y\right)$ and G(y).

The numerical results of the aforesaid statistical tests are obtained by implementing the R-script Adequacy Model with BFGS algorithm.

For a given data set, a probability model with the smallest values of the above statistical tests represents the best-suited model.

**Figure 16:**
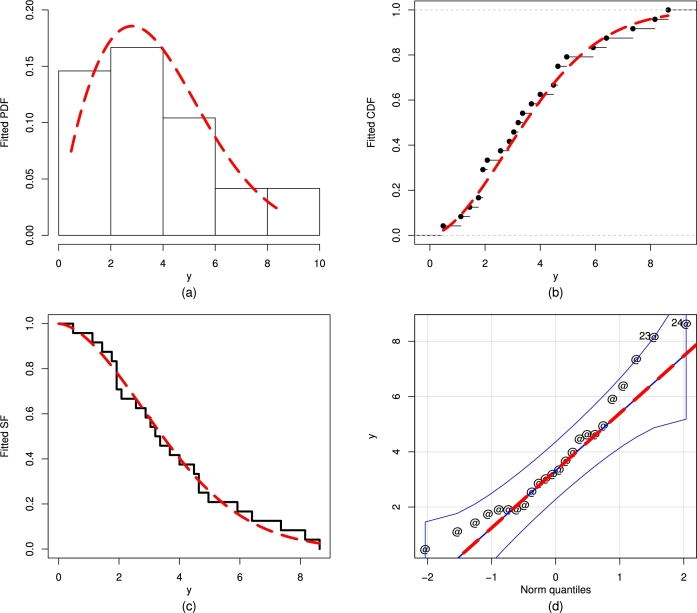
The plots of the (a) fitted PDF, (b) fitted CDF, (c) fitted SF, and (d) QQ function of the UMC-Weibull distribution using the wind energy data.

Using the wind energy data set, the values of the maximum likelihood estimators (MLEs) ${\overset{ˆ}{\tau }}_{MLE}$, ${\overset{ˆ}{\beta }}_{MLE}$, ${\overset{ˆ}{\gamma }}_{MLE}$, ${\overset{ˆ}{\sigma }}_{MLE}$, ${\overset{ˆ}{\eta }}_{MLE}$, and ${\overset{ˆ}{\lambda }}_{MLE}$ of the above competing distributions are reported in Table 2. Furthermore, for the wind energy data set, the comparative results (values of the statistical tests) of the fitted distributions are reported in Table 3. According to the values of $W^{⁎},A^{⁎}$, and $KS^{⁎}$ in Table 3, the best-suited model for the wind energy data set is the UMC-Weibull distribution. Finally, Fig. 16 shows the fitted PDF, SF, QQ, and CDF of the UMC-Weibull distribution. These fitted plots suggest that the UMC-Weibull distribution provides the best-suited fit for the wind energy data set.

**Table 2:** The MLEs of the fitted distributions using the wind energy data recorded on July 19, 2023.

Models	${\overset{ˆ}{\tau }}_{MLE}$	${\overset{ˆ}{\beta }}_{MLE}$	${\overset{ˆ}{\gamma }}_{MLE}$	${\overset{ˆ}{\sigma }}_{MLE}$	${\overset{ˆ}{\eta }}_{MLE}$	${\overset{ˆ}{\lambda }}_{MLE}$
UMC-Weibull	1.71079	0.04823	5.98448	-	-	-
F-Weibull	-	3.00980	-	-	0.18706	-
NMF-Weibull	-	2.22558	-	-	0.19708	1.29659
NCT-Weibull	1.81680	0.00803	-	-	-	-
M-Weibull	1.02653	3.32438	-	3.39113	-	-

**Table 3:** The values of the statistical tests of the fitted distributions using the wind energy data recorded on July 19, 2023.

Models	*W*^⁎^	*A*^⁎^	*KS*^⁎^	p-value
UMC-Weibull	0.02879	0.20579	0.08824	0.99210
F-Weibull	0.03095	0.24337	0.09725	0.97710
NMF-Weibull	0.03078	0.23734	0.09204	0.98710
NCT-Weibull	0.03226	0.22513	0.09830	0.97450
M-Weibull	0.10942	0.35327	0.11150	0.92640

### 4.2 Bivariate analysis of the wind speed and energy data sets

In this section, we apply the BMC-Weibull distribution for analyzing the bivariate wind speed and energy data sets. The wind speed and energy data sets are available at: http://www.sotaventogalicia.com/en/technical-area/real-time-data/historical/. We represent the energy and wind speed data sets by $Y_{1}$ and $Y_{2}$, respectively. Some descriptive measures of the wind speed and energy data sets are presented in Table 4.

**Table 4:** Descriptive measures of the wind speed and energy data sets.

Data Sets	Notation	*n*	Min.	Max.	*Q*_1_	*Q*_2_	*Q*_3_	Mean
Energy	*Y*_1_	24	0.960	17.280	3.840	6.560	9.440	7.547
Wind speed	*Y*_2_	24	5.980	10.440	6.745	7.920	8.795	7.838

#### 4.2.1 Testing of normality of the wind speed and energy data sets

An assessment of the normality of data is a requirement for many statistical tests because normal data is a basic assumption in parametric testing. In this section, we check the wind speed and energy data sets using two well-known statistical tests called the Shapiro-Wilk (SW) normality test and the Anderson-Darling (AD) normality test.

• **The SW normality test**
  In this section, we apply the SW normality test by constructing a hypothesis as follows $H_{0}$: The data is normally distributed vs $H_{1}$: The data is not normally distributed.
  After performing the analysis using the SW normality test, we observe that the SW values of $Y_{1}$ and $Y_{2}$ are given, respectively, by 0.93711 and 0.93957. The p-values corresponding to $Y_{1}$ and $Y_{2}$ are given by 0.1406 and 0.1596. Since the p-values for both $Y_{1}$ and $Y_{2}$ are greater than 0.05, therefore, we can assume the normality of the wind speed and energy data sets.
• **The AD normality test**
  Here, we perform the second normality test of the wind speed and energy data sets using the AD normality test. The AD normality test can be performed by formulating the following hypothesis: $H_{0}$: The data is normally distributed vs $H_{1}$: The data is not normally distributed.
  After carrying out the numerical analysis using the AD normality test, we observe that the AD values of $Y_{1}$ and $Y_{2}$ are given, respectively, by 0.52547 and 0.47317. The p-values corresponding to $Y_{1}$ and $Y_{2}$ are given by 0.1623 and 0.2211. As we can see that the p-values for both $Y_{1}$ and $Y_{2}$ are greater than 0.05, therefore, we fail to reject $H_{0}$ and assume the normality of the wind speed and energy data sets.

#### 4.2.2 Modelling the wind speed and energy data sets

The prime interest of the development of the BMC-Weibull distribution is to be implemented for analyzing in applied sectors. In this section, we illustrate this fact by applying the BMC-Weibull distribution for analyzing the wind speed and energy data sets. The comparison of the BMC-Weibull distribution is made with the

• Farlie–Gumbel–Morgenstern bivariate Weibull (FGMB-Weibull) distribution of Almetwally et al. [32],
  and
• Farlie-Gumble-Morgenstern new heavy-tailed Weibull (FGMNHT-Weibull) distribution of Almaspoor and Tahmasebi [33].

Among the above distributions, the decision about the best-suited distribution for wind speed and energy data sets is made using four statistical criteria. These criteria are given by

• Akaike information criterion (AIC)
  2m−2η(.).
• Bayesian information criterion (BIC)
  mlog⁡(n)−2η(.).
• Hannan-Quinn information criterion (HQIC)
  2mlog⁡[log⁡(n)]−2η(.).
• Consistent Akaike Information Criterion (CAIC)
  $$\frac{2mn}{n-m-1}-2\eta \left(.\right).$$

In the above formulas of information criteria, η(.) denotes the LLF, *m* denotes the number of parameters, and *n* represents the size of the sample. A probability distribution with the largest values of the information criteria is considered the worst performance; while a probability distribution with the smallest values of the information criteria represents the best-suited distribution for the underlined data set. The MLEs and values of the above information criteria for the fitted distributions are obtained using the R-script with library(BB).

Corresponding to the wind speed and energy data sets, the values of ${\overset{ˆ}{\tau }}_{1},{\overset{ˆ}{\tau }}_{2},{\overset{ˆ}{\beta }}_{1},{\overset{ˆ}{\beta }}_{2}$, ${\overset{ˆ}{\gamma }}_{1},{\overset{ˆ}{\gamma }}_{2},{\overset{ˆ}{\theta }}_{1},{\overset{ˆ}{\theta }}_{2}$, and $\overset{ˆ}{\delta }$ of the fitted distributions are provided in Table 5. The values of the considered information criteria of the fitted distributions are provided in Table 6. Corresponding to the given results in Table 6, we can see that the proposed BMC-Weibull distribution has the lowest values of the information criteria. For the BMC-Weibull distribution, the values of the information are given by: AIC = 287.7830, BIC = 262.0300, CAIC = 260.7830, and HQIC = 255.9710. These facts reveal that the BMC-Weibull distribution is the best-suited model for analyzing the wind speed and energy data sets. For the wind speed and energy data sets, the second best-suited model is the FGMNHT-Weibull distribution with AIC = 294.9752, BIC = 267.0131, CAIC = 271.8447, and HQIC = 260.2473.

**Table 5:** The MLEs of the fitted distributions using the wind speed and energy data sets recorded on July 19, 2023.

Models	${\overset{ˆ}{\tau }}_{1}$	${\overset{ˆ}{\tau }}_{2}$	${\overset{ˆ}{\beta }}_{1}$	${\overset{ˆ}{\beta }}_{2}$	${\overset{ˆ}{\gamma }}_{1}$	${\overset{ˆ}{\gamma }}_{2}$	$\overset{ˆ}{\delta }$
BMC-Weibull	3.1940	2.8885	0.0001	0.0009	1.2475	2.3103	0.5343
FGM-Weibull	2.1982	3.6681	0.0085	0.0004	-	-	0.8617
FGMNHT-Weibull	2.6172	3.4607	0.0026	0.0003	3.6318	1.9795	0.7713

**Table 6:** The values of the statistical tests of the fitted distributions using the wind speed and energy data sets recorded on July 19, 2023.

Models	AIC	BIC	CAIC	HQIC
BMC-Weibull	287.7830	262.0300	260.7830	255.9710
FGM-Weibull	295.9280	279.0980	274.3280	264.2650
FGMNHT-Weibull	294.9752	267.0131	271.8447	260.2473

## 5 Concluding remarks

In this paper, two new statistical methods were studied for generating new probability distributions. The first method was called a UMC-*G* family of distributions. It was introduced for generating new univariate probability distributions. Using the proposed UMC-*G*, a new version of the Weibull model called the UMC-Weibull distribution was studied. The effectiveness and application of the UMC-Weibull distribution were shown by analyzing the wind energy data set. The second method was called a BMC-*G* family of distributions. Based on the BMC-*G* method, a bivariate version of the UMC-Weibull model called a BMC-Weibull distribution, was introduced. The BMC-Weibull distribution was applied to the wind speed and energy data sets, and its comparison was made with other bivariate distributions. By considering certain statistical tests, we observed that the BMC-Weibull distribution is the best-suited model for analyzing the wind speed and energy data sets.

In the future, we are motivated to introduce the neutrosophic versions of the UMC-Weibull and BMC-Weibull distributions. Different estimation methods may be incorporated to estimate the parameters of the UMC-Weibull and BMC-Weibull distributions. Furthermore, other trigonometric functions should be considered to introduce new probability distributions for modeling the wind speed and energy data sets.

## CRediT authorship contribution statement

**Badr Alnssyan:** Writing – review & editing, Writing – original draft, Visualization, Validation, Software, Methodology, Investigation, Formal analysis, Data curation, Conceptualization. **Mohammed Ahmed Alomair:** Writing – review & editing, Writing – original draft, Visualization, Validation, Software, Methodology, Investigation, Formal analysis, Data curation, Conceptualization.

## Declaration of Competing Interest

The authors declare no conflict of interest.

## Data Availability

Data included in article/supplementary material/referenced in article.
